# Single Mutation in *iolT1* in *ptsG*-Deficient *Corynebacterium glutamicum* Enables Growth Boost in Xylose-Containing Media

**DOI:** 10.3390/microorganisms13071606

**Published:** 2025-07-08

**Authors:** Katharina Hofer, Lynn S. Schwardmann, Jung-Won Youn, Volker F. Wendisch, Ralf Takors

**Affiliations:** 1 Institute of Biochemical Engineering, University of Stuttgart, 70569 Stuttgart, Germany; katharina.hofer@ibvt.uni-stuttgart.de; 2 Genetics of Prokaryotes, Faculty of Biology and CeBiTec, Bielefeld University, 33615 Bielefeld, Germany; l.schwardmann@web.de (L.S.S.); volker.wendisch@uni-bielefeld.de (V.F.W.); 3 Institute of Microbiology, University of Stuttgart, 70569 Stuttgart, Germany; jung-won.youn@imb.uni-stuttgart.de

**Keywords:** *Corynebacterium glutamicum*, adaptive laboratory evolution, mixed carbon source, *iolT1*, *ptsG*, *iolR*, xylose

## Abstract

Efficient co-utilization of glucose and xylose from lignocellulosic biomass remains a critical bottleneck limiting the viability of sustainable biorefineries. While *Corynebacterium glutamicum* has emerged as a promising industrial host due to its robustness, further improvements in mixed-sugar co-utilization are needed. Here, we demonstrate how a single amino acid substitution can dramatically transform cellular sugar transport capacity. By combining rational strain engineering with continuous adaptive laboratory evolution, we evolved a *ptsG*-deficient *C. glutamicum* strain in glucose–xylose mixtures for 600 h under consistent selection pressure. Whole-genome sequencing revealed a remarkable finding: a single point mutation; exchanging proline for alanine in the *myo*-inositol/proton symporter IolT1 was sufficient to boost glucose uptake by 83% and xylose uptake by 20%, while increasing the overall growth rate by 35%. This mutation, located in a highly conserved domain, likely disrupts an alpha helical structure, thus enhancing transport function. Reverse engineering confirmed that this single change alone reproduces the evolved phenotype, representing the first report of an engineered IolT1 variant in PTS-independent *C. glutamicum* that features significantly enhanced substrate uptake. These results both provide an immediately applicable engineering target for biorefinery applications and demonstrate the power of evolutionary approaches to identify non-intuitive solutions to complex metabolic engineering challenges.

## 1. Introduction

Industrial biotechnology will increasingly rely on sustainable feedstocks to replace petroleum-derived resources and to omit the food versus fuel debate [1,2]. Lignocellulosic biomass, representing one of the most abundant and promising renewable feedstocks, contains glucose and xylose as the most abundant fermentable sugars [3,4]. However, the efficient co-utilization of these mixed sugar streams remains a significant challenge for many industrial microorganisms, limiting the economic viability of bioprocesses utilizing lignocellulosic hydrolysates [5].

*Corynebacterium glutamicum*, a Gram-positive bacterium with GRAS (generally recognized as safe) status widely used for industrial amino acid production [6,7,8], has emerged as an attractive host for biorefinery applications due to its robust metabolism, high tolerance to industrial conditions, and well-established genetic tools [9]. *C. glutamicum* naturally lacks the ability to metabolize xylose. The first breakthrough in engineering xylose utilization was achieved by completing the xylose isomerase (XI) pathway through the integration of the xylose isomerase gene (*xylA*). It was implemented by the heterologous expression of both key genes from *Escherichia coli*—the XI gene (*xylA*) and the xylulokinase gene (*xylB*) [10]—and later improved by varying the XI genes, achieving the fastest growth and production with XI from *Xanthomonas campestris* [11]. This foundational work enabled *C. glutamicum* to convert xylose into the central metabolic pathway via xylulose-5-phosphate, which is introduced into the pentose phosphate pathway.

Over the past decade, extensive engineering efforts focused on developing *C. glutamicum* strains capable of efficiently converting lignocellulosic biomass components into valuable biochemicals [11,12,13,14,15]. Substantial metabolic engineering efforts have been devoted to improving xylose utilization and co-consumption with glucose. These include optimization of xylose transport [12,13,15,16,17], xylose isomerase pathway [11], and downstream pentose phosphate pathway [12,18] components, employing both individual pathway modifications and sophisticated combinatorial engineering strategies targeting multiple bottlenecks simultaneously. A particularly significant discovery emerged from an adaptive laboratory evolution (ALE) study that identified a beneficial mutation in the *ipsA* gene, which encodes a LacI-type transcriptional regulator [14]. Subsequent mechanistic investigations revealed that the IpsA protein can function as a xylose-responsive regulator, directly binding xylose as an effector molecule despite xylose being a non-natural substrate for *C. glutamicum* [14]. Although *C. glutamicum* does not show classical carbon catabolite repression during xylose assimilation, sequential sugar utilization patterns can be observed, which shows that glucose is preferred over xylose [10,13,14,15,17,19,20,21]. This presents a bottleneck for valorizing lignocellulosic feedstocks, as xylose represents the second-most abundant fermentable sugar in biomass hydrolysates [22].

Additionally, the phosphoenolpyruvate (PEP) dependency of the PTS (phosphotransferase system) for glucose uptake can limit the availability of this crucial metabolic intermediate for biosynthetic pathways, particularly if PEP is required as a precursor [5,13,23]. Alternatively, decoupling glucose uptake from the PTS can be achieved by the uptake and subsequent phosphorylation of glucose by the *myo*-inositol/proton symporter IolT1 or IolT2 and the ATP-dependent glucokinase (Glk) or polyphosphate/ATP-dependent glucokinase (PpgK) [23]. IolT1 has also been identified to contribute to xylose uptake in *C. glutamicum* [24]. However, IolT1 expression is normally repressed by the transcriptional regulator IolR, limiting its contribution to sugar transport [24].

While rational engineering approaches have provided important insights, they often fail to identify optimal solutions due to the complexity of cellular metabolism and regulatory networks. ALE offers a complementary strategy that harnesses natural selection to obtain desired phenotypes [25]. Potential beneficial mutations that may not be apparent through rational design can be identified by sequencing and subsequently verified through reverse engineering.

In this study, we combined rational strain engineering with continuous ALE to develop an improved *C. glutamicum* strain capable of efficient parallel glucose and xylose co-utilization. Starting with a *ptsG*-deficient strain engineered for xylose metabolism, we deleted *iolR* to enhance IolT1-mediated transport of glucose and xylose. The resulting strain was subjected to prolonged continuous cultivation in glucose–xylose mixtures. Whole-genome sequencing of evolved populations revealed several interesting mutations that resulted in enhanced growth and uptake rates in mixed sugar cultivations. Subsequent reverse engineering verified a single point mutation in *iolT1* that dramatically enhances both glucose uptake (+83%) and xylose uptake (+20%) rates. These results provide new insights into *iolT1* engineering and demonstrate the power of combining rational and evolutionary approaches for strain improvement.

## 2. Materials and Methods

### 2.1. Bacterial Strains

All bacterial strains used in this study are listed in Table 1.

### 2.2. Recombinant DNA Work

The plasmids and oligonucleotides used in this study are listed in Supplementary Tables S1 and S2. Plasmid construction and amplification were performed in *E. coli* DH5α [26]. *E. coli* strains were grown at 37 °C and 180 rpm in lysogeny broth (LB; 5 g L^−1^ yeast extract, 10 g L^−1^ tryptone, 10 g L^−1^ NaCl [30]) supplemented with kanamycin (50 µg mL^−1^). Chemically competent *E. coli* cells [30] were transformed by heat shock at 42 °C [31].

The genomic DNA (gDNA) of *C. glutamicum* strains was isolated using a commercially available kit (NucleoSpin Microbial DNA Mini kit for DNA from microorganisms, Macherey-Nagel, Düren, Germany) or by salt precipitation [32]. Plasmid isolation, cloning, and transformation were performed as described previously [27,32]. All enzymes (Q5 High-Fidelity DNA Polymerase, Antarctic Phosphatase, XbaI, HindIII, and SmaI) were purchased from NEB (New England Biolabs GmbH, Frankfurt, Germany), and the listed oligonucleotides for DNA amplification and sequencing (Supplementary Table S2) were acquired from Metabion International AG (Planegg/Steinkirchen, Germany) and Eurofins Genomics (Ebersberg, Germany).

The strains *C. glutamicum* gXP, *C. glutamicum* gXPΔ*iolR*, and *C. glutamicum* gXPΔ*iolR*Δ*iolT1* were constructed by double homologous recombination for markerless gene deletion, using the suicide vector pK19*mobsacB* [33]. The same vector and strategy were adopted for the genomic integration of the mut*iolT1* gene in the *iolT1* locus, yielding the strain *C. glutamicum* gXPΔ*iolR*Δ*iolT1*::mut*iolT1*.

For homologous recombination, the flanking genomic regions of deletion or integration sites were amplified from *C. glutamicum* WT gDNA, as described previously [20], using oligonucleotides UF- and DF-fw and -rv. The plasmid pK19*mobsacB* was linearized by restriction with XbaI/HindIII or SmaI. Purified DNA fragments and dephosphorylated, linearized plasmids were joined via Gibson Assembly [34]. The insert sequences and correct assembly of all constructed plasmids were confirmed by Sanger sequencing.

Genomic deletions of the *C. glutamicum* strains gXP and gXPΔ*iolR* were performed by conjugation using the donor strain *E. coli* S17-1 following a routine protocol, while electroporation was performed for the introduction of the deletion of *iolT1* and the subsequent insertion of mut*iolT1* in the strains *C. glutamicum* gXPΔ*iolR*Δ*iolT1* and *C. glutamicum* gXPΔ*iolR*Δ*iolT1*::mut*iolT1* [31]. All deletions and the integration of mut*iolT1* were verified by Sanger sequencing with the respective oligonucleotides UF-g and DF-g.

### 2.3. Preculture and Media

*C. glutamicum* strains were cultivated in modified CgXII medium [35], which contained 20 g L^−1^ (NH_4_)_2_SO_4_, 5 g L^−1^ urea, 1 g L^−1^ KH_2_PO_4_, 1 g L^−1^ K_2_HPO_4_, 21 g L^−1^ 3-(N-morpholino)propanesulfonic acid (MOPS), 0.25 g L^−1^ MgSO_4_ × 7H_2_O, 0.01 g L^−1^ CaCl_2_, 0.2 mg L^−1^ D-biotin, 0.313 g L^−1^ CuSO_4_ × 5H_2_O, 16.4 mg L^−1^ FeSO_4_ × 7H_2_O, 10 mg L^−1^ MnSO_4_ × H_2_O, 0.02 mg L^−1^ NiCl_2_ × 6H_2_O, and 1 mg L^−1^ ZnSO_4_ × 7H_2_O. Changes in the medium are indicated. Precultures were grown in LB or double yeast tryptone medium (dYT; 16 g L^−1^ tryptone, 10 g L^−1^ yeast extract, 5 g L^−1^ NaCl [30]). The detailed preculture procedure and cultivation conditions are mentioned in the following.

For BioLector micro-cultivation, precultures were inoculated from fresh LB agar plates (18 g L^−1^ agar) and grown in 5 mL liquid LB at 30 °C for 8 h under constant agitation (120 rpm) on a benchtop rotary shaker (Infors HT, Bottmingen, Switzerland). The cultures for growth experiments were inoculated to an optical density at 600 nm (OD_600_) of 1, using overnight precultures, which were washed before the inoculation of the main culture.

For the continuous adaptive evolution and subsequent shake flask cultivations, 20 µL of glycerol (30% *v*/*v*) stocks of the respective *C. glutamicum* strain was used to inoculate 5 mL dYT medium. The glass cultivation tube was incubated for 8 h at 30 °C under constant agitation (120 rpm) on a benchtop rotary shaker (Infors HT, Bottmingen, Switzerland). The preculture was used to inoculate 50 mL of modified minimal medium CgXII supplemented with 15.34 g L^−1^ glucose and 4.66 g L^−1^ xylose in 500 mL baffled shake flasks and incubated under the aforementioned conditions for 12–14 h.

### 2.4. BioLector Micro-Cultivations

The growth performance of *C. glutamicum* strains in modified CgXII medium supplemented with 42 g L^−1^ MOPS, 30 mg L^−1^ protocatechuic acid (PCA), and 40 g L^−1^ glucose or xylose was performed in a BioLector micro-cultivation system in a 48-well flower plate (m2p-labs, Aachen, Germany). The cultivation volume amounted to 1 mL with a starting OD_620_ of 1. The cells were grown at 30 °C, 85% humidity, and 1100 rpm. OD_620_ was recorded automatically.

### 2.5. Continuous Adaptive Laboratory Evolution

A continuous laboratory adaptive evolution of the strain *C. glutamicum* gXP Δ*iolR* was performed in a glass bioreactor, with a working volume of 0.5 L. The reactor was equipped with a six-blade Rushton impeller, a pH probe, and a dissolved oxygen (DO) probe (Mettler Toledo GmbH, Albstadt, Germany). Temperature and pressure were kept constant at 30 °C and 1.5 bar, respectively. A pH of 7.4 was maintained by automatically drip-feeding 25% (*v*/*v*) NH_4_OH solution. Oxygen saturation was kept above 30% by increasing the impeller speed in steps of 10 rpm. The batch cultivation was initiated with a starting OD_600_ of 1 and a volume of 0.5 L. Five percent (*v*/*v*) was harvested from the preculture shake flasks, centrifuged at 4000× *g* (5430 R, Eppendorf, Hamburg, Germany) at 4 °C for 5 min, washed, and resuspended in 10 mL sterile 0.9% (*w*/*v*) NaCl solution. It was then used to inoculate CgXII minimal medium supplemented with 7.73 g L^−1^ glucose and 2.23 g L^−1^ xylose. Urea and MOPS were omitted. When necessary, an antifoam agent (Struktol^®^ J 647, Schill + Seilacher, Hamburg, Germany) was added manually. At the end of the batch phase, which was indicated by a sudden rise in the DO signal, the stirrer speed was set to 470 rpm and the aeration rate to 0.5 vvm. Feed and harvest pumps were started, and the pumping speed was adjusted to reach a dilution rate of 0.1 h^−1^ in the first stage of the continuous cultivation and a dilution rate of 0.2 h^−1^ in the following stage. During the continuous cultivation, 60 µL h^−1^ of antifoam agent was dosed constantly via a syringe pump (LA-30, Landgraf HLL, Langenhagen, Germany). The feed medium composition was identical to the batch medium. After 300 h, the first stage of the continuous adaptive evolution was stopped, and cell suspension was harvested aseptically and frozen at −70 °C with 30% (*v*/*v*) glycerol. This aliquot was used to start the second stage of the continuous adaptive evolution with a preceding batch phase, as described before. The second stage of the continuous adaptive evolution lasted for an additional 300 h. A schematic overview of the ALE process can be found in the Supplementary Material (Supplementary Figure S1).

### 2.6. Shake Flask Cultivations

Shake flask cultivations were performed in triplicate in baffled 500 mL shake flasks (DWK Life Sciences, Wertheim, Germany) with a liquid volume of 50 mL. The amount of biomass needed to inoculate the main culture with an initial OD_600_ of 1 was harvested from the overnight precultures, centrifuged at 5000 rpm (5430 R, Eppendorf, Hamburg, Germany) at 4 °C for 5 min, washed, and resuspended in 1 mL sterile 0.9% (*w*/*v*) NaCl solution. The resulting cell suspension was used to inoculate 50 mL modified CgXII minimal medium. For the xylonate accumulation experiment, the medium was additionally supplemented with 30 mg L^−1^ PCA, and three different carbon source compositions were tested: (1) 15 g L^−1^ glucose + 5 g L^−1^ xylose, (2) 5 g L^−1^ xylose only and (3) 20 g L^−1^ xylose only. We considered the ratio of glucose to xylose 3:1 to be a characteristic proportion representative of typical lignocellulosic hydrolysates. For the characterization of the reverse engineered strain, the medium contained 15.34 g L^−1^ glucose and 4.66 g L^−1^ xylose. The cultures were incubated at 30 °C under constant agitation (120 rpm) on a benchtop rotary shaker (Infors HT, Bottmingen, Switzerland) for up to 72 h.

### 2.7. Analytical Methods and Whole-Genome Sequencing

During shake flask cultivations, bacterial growth was monitored by the measurement of OD_600_ (DR 3900, Dr. Lange, Berlin, Germany). Cell-free samples were produced by centrifuging 0.5 mL of cell suspension at 4000× *g* at 4 °C for 5 min. The cell-free supernatant was stored at −20 °C until measurement. Generally, glucose, xylose, and organic acids were determined by HPLC using the Agilent 1200 series system (Agilent Technologies, Waldbronn, Germany), which was equipped with a Hi-Plex H column (7.7 × 300 mm, 8 μm) and a Hi-Plex H guard cartridge (3 × 5 mm, 8 μm). The column temperature was 50 °C. Samples were diluted if necessary and eluted isocratically with a flow rate of 0.4 mL min^−1^ for 35 min using 5 mM H_2_SO_4_ as a mobile phase. Analytes were detected using a refractive index detector (RID). Since the coelution of xylose and xylonate occurring during the xylonate accumulation experiment prohibited clear differentiation, a diode array detector (DAD G1315B, 1200 series, Agilent Technologies) was used for the detection of xylonate at 210 nm, while xylose concentrations of corresponding samples were determined by an enzymatic assay (D-xylose assay kit, Megazyme Ltd., Wicklow, Ireland).

During continuous cultivations, bacterial growth was monitored by the measurement of OD_600_ and the determination of cell dry weight (CDW). For the latter, 1 mL cell suspension was harvested and washed twice in deionized water, centrifuging between the washing steps at 20,173× *g* and 4 °C for 4 min. The washed biomass was transferred to pre-weighed glass vials and dried for 48–72 h in a convection oven (Heraeus, Hanau, Germany) at 105 °C. After cooling to room temperature in a desiccator, the vials containing the dried biomass were weighed on a micro-scale (AE 200, Mettler Toledo, Gießen, Germany). Cell-free samples were produced by centrifuging 0.5 mL of cell suspension at 4000× *g* and 4 °C for 5 min. The cell-free supernatant was stored at −20 °C until analysis. Glucose and xylose concentrations were determined with enzymatic assays following the manufacturer’s instructions (R-biopharma, Darmstadt, Germany, and Megazyme, Bray, Ireland).

For whole-genome sequencing (WGS) samples, suspension was harvested aseptically and frozen at −70 °C with 30% (*v*/*v*) glycerol. DNA extraction, WGS, and subsequent single nucleotide polymorphism (SNP)/Indel analysis were performed by Azenta Life Sciences, Leipzig, Germany. Illumina NovaSeq 2 × 150 bp sequencing was performed.

### 2.8. Determination of Kinetic Parameters

All equations for the calculation of kinetic parameters are listed in Supplementary Table S3. During batch cultivations (bioreactor and shake flask), the exponential growth rate (μ_max_) was determined by the linear regression of the logarithmic biomass concentration over the respective time. Given that steady states were achieved during continuous operation, the set dilution rate (D) equals the growth rate µ [35].

Biomass-substrate yields (Y_X/S_) were determined by the linear regression of substrate and biomass concentration curves during the batch phase. During continuous cultivation, the biomass substrate yield was calculated with steady-state concentrations. Biomass-specific substrate consumption rates for glucose and xylose (q_glc_ and q_xyl_) of the strains were calculated by dividing the determined exponential growth rate (μ_max_) or the set dilution rate D by Y_X/S_.

## 3. Results

### 3.1. Strain Engineering

**Figure 1:**
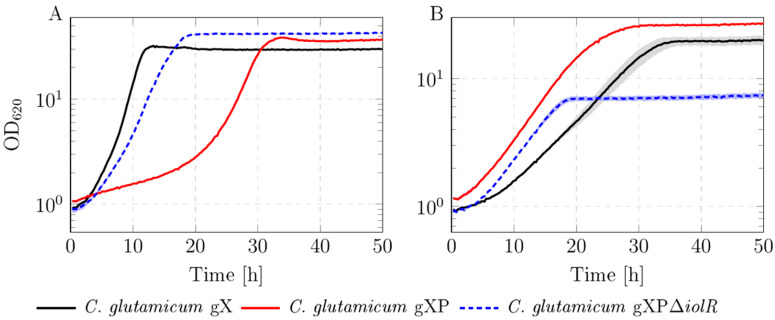
Growth analysis of the strains *C. glutamicum* gX, *C. glutamicum* gXP, and *C. glutamicum* gXPΔ*iolR* in (**A**) modified CgXII medium supplemented with 40 g L^−1^ glucose and (**B**) modified CgXII medium supplemented with 40 g L^−1^ xylose. Strain screening was performed in a BioLector micro-cultivation system. Data represent means of 3 replicates with standard deviations as error bands (shaded areas).

The xylose-utilizing strain *C. glutamicum* gX [20] was used as a starting point for further improvements. In this study, we aimed to improve *C. glutamicum* gX regarding substrate uptake rates and growth rate. Growing the *ptsG*-deficient *C. glutamicum* gXP revealed a lag phase in glucose, but not in xylose-containing medium compared with *C. glutamicum* gX (Figure 1A). Interestingly, no lag phase occurred upon additional deletion of *iolR* (strain *C. glutamicum* gXPΔ*iolR*) that showed derepressed glucose and xylose uptake by IolT1 due to the deleted gene *iolR* [24]. While growth in glucose-containing medium was restored, biomass formation was strongly impaired when xylose was the sole carbon source (Figure 1B).

### 3.2. Impact of Substrate Composition on Xylose Utilization and Xylonate Accumulation

**Figure 2:**
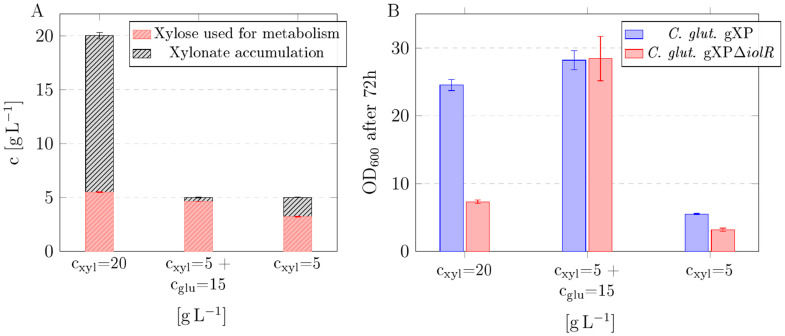
Shake flask cultivation of *C. glutamicum* gXP and *C. glutamicum* gXPΔ*iolR* under different substrate conditions. (**A**) Absolute concentrations of xylose used for metabolism in *C. glutamicum* gXPΔ*iolR* and resulting xylonate accumulation after 72 h of cultivation. Conditions include high xylose concentration (c_xyl_ = 20 g L^−1^), mixed substrate (c_xyl_ = 5 g L^−1^ + c_glc_ = 15 g L^−1^), and low xylose concentration (c_xyl_ = 5 g L^−1^). (**B**) Comparison of *C. glutamicum* gXP and *C. glutamicum* gXPΔ*iolR*: change in OD_600_ within 72 h represents net biomass accumulation. Data represent means of 3 replicates with standard deviations as error bars.

The oxidation of xylose to xylonic acid (xylonate) and the accumulation thereof in *C. glutamicum* Δ*iolR* was previously shown [19]. To investigate the effect of substrate composition on xylose metabolism and xylonate formation in *C. glutamicum* gXPΔ*iolR*, cultures were supplemented with either two different concentrations of xylose alone or a mixture of xylose and glucose. The resulting concentrations of xylose, accumulated xylonate, and net growth after 72 h of cultivation are presented in Figure 2. In cultures with a high xylose concentration of 20 g L^−1^, a substantial portion of the substrate was diverted towards xylonate accumulation, with only around 27% of the xylose utilized for metabolic purposes (Figure 2A). In contrast, the mixed substrate condition (5 g L^−1^ xylose + 15 g L^−1^ glucose) resulted in negligible xylonate accumulation, with almost all xylose channeled into metabolism. Interestingly, cultures grown with a low xylose of 5 g L^−1^ as the sole carbon source showed a moderate xylonate accumulation of around 1.5 g L^−1^, with the remaining xylose (around 65%) used for metabolism. For investigating the substrate conversion efficiency of *C. glutamicum* gXPΔ*iolR*, growth was compared with *C. glutamicum* gXP (Figure 2B). *C. glutamicum* gXPΔ*iolR* metabolized the largest fraction of xylose (>93%) under mixed substrate conditions and showed comparable biomass production to *C. glutamicum* gXP. The supply of high xylose concentrations resulted in inefficient substrate utilization and maximal xylonate overflow (Figure 2A,B) in *C. glutamicum* gXPΔ*iolR*. A low xylose concentration without co-feeding of glucose led to a moderate metabolic efficiency in *C. glutamicum* gXPΔ*iolR* (Figure 2A,B). In summary, a very high xylose supply triggered xylonate accumulation, while glucose co-feeding promoted efficient xylose utilization.

### 3.3. Continuous Adaptive Laboratory Evolution

**Figure 3:**
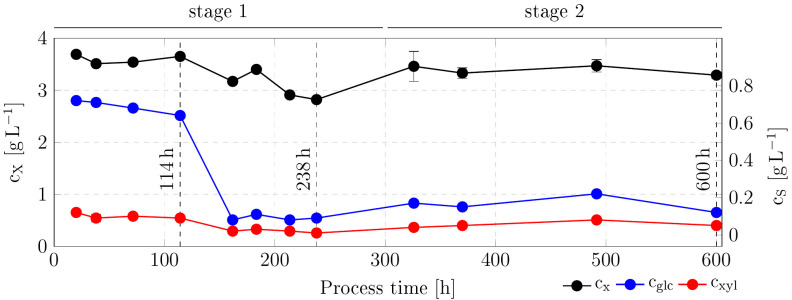
Overview of the continuous adaptive laboratory evolution of *C. glutamicum* gXPΔ*iolR*. Stage 1 lasted from 0 to 300 h operated with a dilution rate D of 0.10 ± 0.00 h^−1^. Stage 2 was subsequently started with a dilution rate D set to 0.21 ± 0.01 h^−1^. Biomass concentration (c_x_) and extracellular glucose (c_glc_) and xylose (c_xyl_) concentrations (all in g L^−1^) over process time are shown. Relevant time points are indicated by vertical dashed lines at respective process times of 114, 238, and 600 h. Two potentially evolved subpopulations were harvested for subsequent WGS: *C. glutamicum* gXPΔ*iolR* ALE_1 (in the following ALE_1) after 238 h of continuous ALE and *C. glutamicum* gXPΔ*iolR* ALE_2 (in the following ALE_2) after 600 h of continuous ALE. All data represent means of 3 measurements with standard deviations as error bars.

Two consecutive continuous bioreactor cultivations were performed to obtain *C. glutamicum* gXPΔ*iolR* populations with improved sugar uptake rates and an increased growth rate. The medium was chosen to select for the subpopulation with the desired traits: CgXII minimal medium supplemented with glucose and xylose without the addition of PCA. PCA-free medium was specifically selected to reduce medium costs. The course of the process is given in Figure 3. The total process time of 600 h refers to the sum of the two consecutive cultivations. The first cultivation started with the parental strain *C. glutamicum* gXPΔ*iolR* and a set dilution rate of D = 0.10 ± 0.00 h^−1^. After 114 h of cultivation time, the extracellular glucose and xylose concentrations in the reactor decreased, indicating a change in the metabolism of the sub-population present in the bioreactor at that time point (Figure 3, first vertical dashed line). After 300 h, the first process was stopped to recalibrate the equipment and prevent fouling of the pH and DO probes. The subsequent process was initialized as previously described using the final subpopulation of the preceding process as inoculum. However, for the second stage of continuous ALE, the dilution rate D was increased to 0.21 ± 0.01 h^−1^, ensuring stable but faster process conditions. After a total process time of 600 h (25 days), the process was stopped. Over the course of the two consecutive continuous cultivations, two potentially evolved subpopulations were harvested for whole-genome sequencing (WGS): *C. glutamicum* gXPΔ*iolR* ALE_1 (in the following ALE_1) was harvested after 238 h of continuous cultivation (Figure 3, second vertical dashed line), and *C. glutamicum* gXPΔ*iolR* ALE_2 (in the following ALE_2) after 600 h of continuous cultivation (Figure 3, third vertical dashed line).

**Figure 4:**
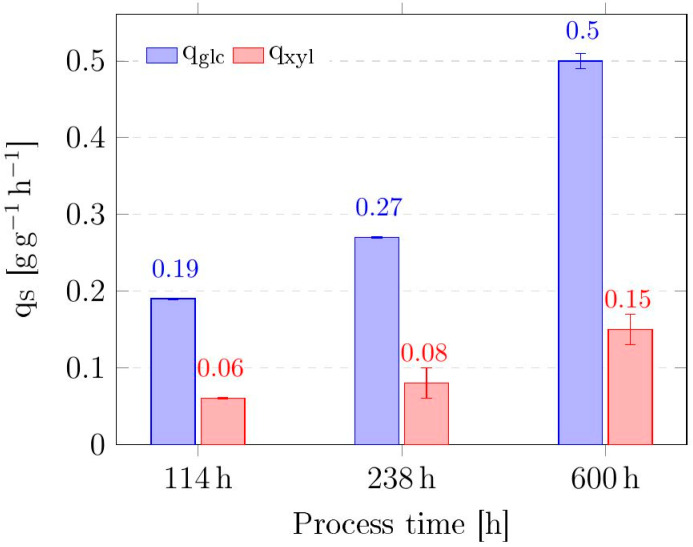
Biomass-specific uptake rates q_s_ in g g^−1^ h^−1^ of *C. glutamicum* gXPΔ*iolR* for the two substrates glucose (glc) and xylose (xyl) at relevant time points throughout the continuous adaptive evolution. Data represent means of 3 measurements with standard deviations as error bars.

To evaluate changes in substrate uptake, biomass-specific uptake rates of glucose and xylose were calculated for relevant time points (Figure 4). Within the first stage of the process, between 114 and 238 h of continuous ALE, the biomass-specific uptake rates for glucose (q_glc_) and xylose (q_xyl_) increased by 42% and 33%, respectively. In stage 2 (600 h), q_glc_ and q_xyl_ increased further by 85% and 88%, respectively. In other words, q_glc_ and q_xyl_ raised 2.6- and 2.5-fold compared with the initial status. Surprisingly, the ratio between the biomass-specific xylose and glucose uptake rates did not change, but leveled at around 0.3 for all quantification time points.

**Figure 5:**
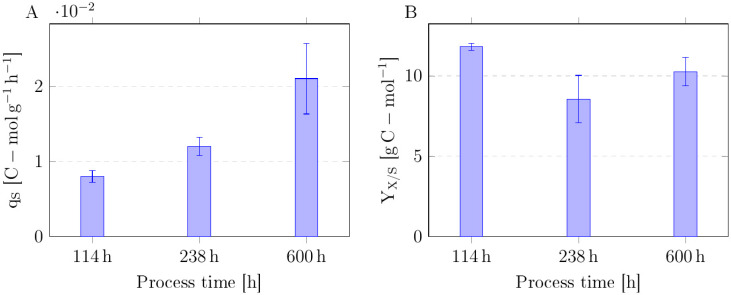
(**A**) Biomass-specific molar carbon uptake rates q_s_ in C-mol g^−1^ h^−1^ and (**B**) biomass substrate yield Y_X/S_ in g C-mol^−1^ *C. glutamicum* gXPΔ*iolR* at relevant time points throughout the adaptive evolution. Data represent means of 3 independent measurements with standard deviations as error bars.

To study metabolic changes, biomass-specific carbon uptake rates and substrate-to-biomass yields were calculated on a molar basis (Figure 5A,B). During ALE, the biomass-specific molar carbon uptake rate of *C. glutamicum* gXPΔ*iolR* increased from 0.008 ± 0.001 C-mol g^−1^ h^−1^ to 0.021 ± 0.005 C-mol g^−1^ h^−1^. The biomass substrate yield decreased from 11.81 ± 0.22 g C-mol^−1^ to 8.55 ± 1.48 g C-mol^−1^ in the first stage of the process, but was nearly restored within the second stage of the process and amounted to 10.26 ± 0.89 g C-mol^−1^ after 600 h of cultivation (Figure 5B).

### 3.4. Mutations in Evolved Populations ALE_1 and ALE_2

Whole-genome sequencing of the populations ALE_1 and ALE_2 was performed to identify genomic mutations that potentially occurred during the continuous ALE and could explain the observed phenotype (Table 2 and Table 3). The population ALE_1 displayed a mutation in the *iolT1* gene (cg0223), encoding the *myo*-inositol/proton symporter IolT1, where a base exchange led to an amino acid exchange from alanine to proline at position 452, referred to as IolT1^A452P^ in the following. Furthermore, a base exchange in the 16s rRNA copy E at position 2,689,275 was found (Table 2). These two mutations were rediscovered in the population ALE_2 (Table 3). In addition, ALE_2 featured mutations in the *dtxR* gene (cg2103), encoding the iron-dependent regulator DtxR, where arginine was replaced by histidine at position 103. In the gene *ipsA* (cg2910) encoding the HTH (helix-turn-helix)-type transcriptional regulator IpsA, aspartic acid was replaced by asparagine at position 141. In ALE_2, another mutation in the 16s rRNA copy E was found, which is adjacent to the previously found mutation in the 16s rRNA copy E in ALE_1. All identified mutations were detected in the genomic DNA of the whole-population samples ALE_1 and ALE_2 (rather than individual clones), each with a frequency above 90%.

### 3.5. Reverse Engineering of One Mutation into Parental Background and Growth Characterization

**Figure 6:**
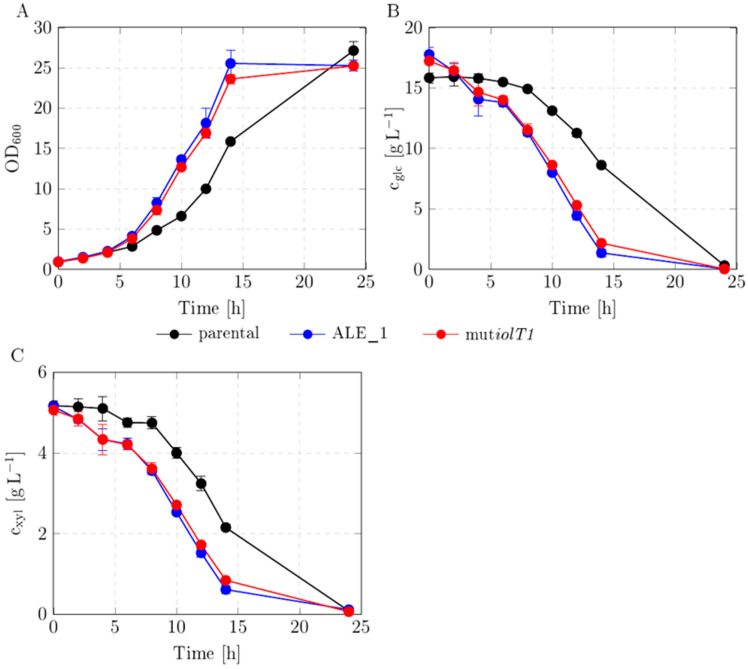
Growth behavior and sugar uptake rates of parental *C. glutamicum* gXPΔ*iolR*, evolved *C. glutamicum* gXPΔ*iolR* (ALE_1), and reverse-engineered *C. glutamicum* gXPΔ*iolR* (mut*iolT1*) strains. Cultivations were conducted in modified CgXII medium supplemented with glucose and xylose. (**A**) Growth was monitored by the measurement of OD_600_. (**B**) Extracellular glucose concentrations (c_glc_) and (**C**) extracellular xylose concentrations (c_xyl_) were determined for each indicated measurement time point. Data represent means of 3 replicates with standard deviations as error bars.

To evaluate and verify the phenotypic effect, the mutation IolT1^A452P^ was reverse-engineered into the parental background *C. glutamicum* gXPΔ*iolR*. The resulting strain *C. glutamicum* gXPΔ*iolR*mut*iolT1* (also referred to as mut*iolT1*) was benchmarked against ALE_1 and the parental background strain regarding growth rate and sugar uptake rates in modified CgXII medium supplemented with glucose and xylose. The reverse-engineered strain *C. glutamicum* gXPΔ*iolR*mut*iolT1* exhibited growth kinetics and sugar uptake kinetics comparable to ALE_1 (Figure 6 and Table 4). The kinetic parameters for each of the three strains were calculated and are listed in Table 4. The parental strain exhibited a growth rate μ of 0.20 ± 0.00 h^−1^, whereas both the ALE_1 and the mut*iolT1* strain were characterized by significantly increased (*p* ≤ 0.001) growth rates of 0.27 ± 0.01 h^−1^, an increase of 35%. The glucose uptake rate (q_glc_) of mut*iolT1* significantly increased by 83% (*p* ≤ 0.001), and xylose consumption (q_xyl_) was 20% (*p* ≤ 0.05) higher compared with the parental background. Both the evolved strain ALE_1 and the reverse-engineered strain *C. glutamicum* gXPΔ*iolR* mut*iolT1* showed similar growth and substrate uptake kinetics, highlighting the phenotypic effect on metabolism by this single mutation.

## 4. Discussion

### 4.1. Deletion of ptsG Leads to Better Growth in Xylose-Containing Medium

This study was motivated by the aim of further improving xylose and glucose uptake and consumption thereof in *ptsG*-negative *C. glutamicum* cultivated in medium with mixed carbon sources. In the first step, the *ptsG* gene was deleted in the xylose-utilizing strain *C. glutamicum* gX [20], yielding *C. glutamicum* gXP. In this strain, PTS-independent glucose uptake and utilization is achieved by IolT1/IolT2 followed by phosphorylation via the kinases PpgK or Glk [23]. Simultaneously, xylose is also taken up by IolT1 [24] and further metabolized via *xylA* and *xylB* [10]. Compared with *C. glutamicum* gX, *C. glutamicum* gXP showed reduced growth in glucose-containing medium (Figure 1A). In xylose-containing medium, however, the growth rate increased after the deletion of *ptsG* (Figure 1B). Wang et al. introduced *xylA* and *xylB* genes from *E. coli* K-12 into the *pta-ackA* (phosphotransacetylase-acetate kinase) locus and observed that *ptsG* deficiency in their *C. glutamicum* mutant caused noticeably reduced growth in medium containing xylose as the sole carbon source compared with the *ptsG*-harboring strain. Our data show that the introduction of *xylA* and *xylB* into the *actA* locus, which was performed in *C. glutamicum* gX [20], is more beneficial for growth in xylose in *ptsG*-negative *C. glutamicum* strains (here, *C. glutamicum* gXP). This improved performance can be attributed to the differential regulation of these loci: while *actA* is known to be expressed strongly and constitutively [36], the *ack* and *pta* genes are subject to regulation by various transcriptional regulators [37]. Thus, integration into the *actA* locus is preferable over integration into *pta-ackA* unless all regulatory sequences of the latter locus have been deleted.

In the next step, we deleted the gene *iolR* encoding the GntR-type transcriptional repressor IolR [38] in *C. glutamicum* gXP, leading to *C. glutamicum* gXPΔ*iolR*. The consequent derepression of the glucose and xylose transporter IolT1 [23] led to the improved uptake of the two sugars. Thus, *C. glutamicum* gXPΔ*iolR* was characterized by restored growth in glucose-containing medium (Figure 1B). With xylose as the sole carbon source, this strain showed greatly decreased biomass formation (Figure 1A), which can be explained by the withdrawal of xylose from use for metabolism by the formation of xylonate. The endogenous *myo*-inositol dehydrogenase IolG is the main enzyme responsible for the oxidation of xylose to xylonate [19]. The deletion of the *iolR* gene in *C. glutamicum* gXPΔ*iolR* leads to the increased transcription of *iolG* [38] and explains the xylonate accumulation in this strain. We demonstrated this by cultivating the strains *C. glutamicum* gXP and *C. glutamicum* gXPΔ*iolR* under different substrate conditions. The data show that glucose co-feeding markedly enhanced metabolic xylose utilization and suppressed xylonate overflow. As shown in Figure 2A, when grown in medium containing xylose as the sole carbon source at a high concentration of 20 g L^−1^, the majority of the xylose is oxidized to xylonate in the strain *C. glutamicum* gXPΔ*iolR* despite the presence of functional *xylA* and *xylB*. In summary, deleting *iolR* alleviated the repression not only of IolT1 but also of the *myo*-inositol dehydrogenase IolG [19]. Interestingly, when xylose was present at a lower concentration of 5 g L^−1^, xylonate formation was significantly reduced and co-feeding of 15 g/L glucose even minimized xylose depletion towards xylonate to 7% (Figure 2A). This indicates a competition between the isomerase pathway (*xylA*/*xylB*) and the oxidation of xylose to xylonate. The utilization of a mixture of glucose and xylose is particularly relevant as it mimics the sugar composition of lignocellulosic hydrolysates, containing glucose and xylose as major components [3]. Efficient co-utilization of these sugars is one of the key prerequisites for the biotechnological valorization of these renewable feedstocks. For this reason, the next stage was to improve the strain *C. glutamicum* gXPΔ*iolR* in medium containing both glucose and xylose via ALE and identify the mutations responsible for an improved phenotype.

### 4.2. Carbon Uptake Dynamics During ALE Cultivation Reveal Improved Co-Utilization with Stable Sugar Preference

In our study, we employed a continuous ALE approach rather than traditional repetitive batch cultivations [39,40,41] to enhance both glucose and xylose utilization in *C. glutamicum* gXPΔ*iolR* in protocatechuic acid (PCA)-free medium. The continuous approach offered the advantage of a consistent and clearly defined selection pressure throughout the evolution experiment [35], where two stable levels could be set that differed by dilution rate. In contrast, batch-wise ALE approaches subject cells to highly fluctuating growth environments regarding substrate and biomass concentrations, oxygen availability, and pH shifts over process time [25]. Such variability can introduce unintended selection pressures that may obscure the desired selection target. Moreover, repetitive batch ALE typically maintains high sugar concentrations throughout most of the cultivation period, primarily selecting for maximum growth rates under substrate-saturated conditions. In contrast, continuous ALE maintains low, steady-state substrate concentrations, enabling simultaneous selection for both enhanced growth rates and improved substrate affinity [42]. The approach presented here was explicitly selected for the best-growing subpopulations, while slower-growing variants were washed out. This achieves direct selection of improved strains rather than those that might excel at competition in nutrient- or oxygen-depleted environments. The continuous mode also prevented stationary phases that might occur during batch-wise selection approaches [43], maintaining cells in a more consistent physiological state throughout the experiment. Additionally, the iron chelator PCA, a costly medium component typically added to enhance iron acquisition, was omitted. While PCA supplementation has been shown to boost the growth of *C. glutamicum* [44], its elimination represents an important step toward developing more cost-efficient industrial processes. A previously published continuous ALE experiment was successfully conducted, selecting for fast-growing subpopulations in the absence of the expensive supplement PCA [35].

Throughout the continuous ALE, the uptake rates of both glucose and xylose of the evolved *C. glutamicum* gXPΔ*iolR* increased markedly, suggesting that the uptake system of both sugars was subject to positive selection pressure. Notably, the ratio of xylose to glucose uptake remained relatively constant at approximately 0.3 across all time points. This implies that the parental strain *C. glutamicum* gXPΔ*iolR* was able to co-utilize glucose and xylose from the beginning and that the cells kept the initial carbon preference. A recent publication showed that evolved and engineered *C. glutamicum* is able to co-consume xylose and glucose with a ratio of up to around 0.6 [14]. The parental strain of this study, *C. glutamicum* CGS6, acquired co-consumption capabilities only during ALE [14]. By contrast, our data show that the deletion of *ptsG* together with the introduction of *xylAB* already enabled the co-utilization of glucose and xylose in *C. glutamicum.*

Graf et al. identified the maximum metabolic capacity q_C_ of *C. glutamicum* ATCC13032, with 0.0216 ± 0.0015 Cmolg^−1^ h^−1^ in pure glucose [45]. Interestingly, ALE_2 almost achieved this maximum with 0.021 ± 0.005 Cmolg^−1^ h^−1^ having started at 0.008 ± 0.001 Cmolg^−1^ h^−1^ (Figure 5A). The observed biomass-carbon yield Y_XS_ of the ALE_2 population in the applied glucose–xylose mixture was around 30% lower than that published for *C. glutamicum* pEKEx3-xyl*XABCD*_Cc_ engineered for xylose utilization via the alternative Weimberg pathway [46]. Although the Weimberg pathway is characterized by reduced carbon loss during xylose utilization [46], this might indicate the formation of by-products in ALE_2. During faster growth, to avoid metabolic bottlenecks and to maintain redox balance, excess carbon was directed into the production of side-products like lactate, acetate, and succinate.

### 4.3. Preliminary Characterization of iolT1 Mutation Reveals Growth and Uptake Boost

A total of five genomic mutations were found in the ALE_2 population, two of which were already present in the ALE_1 population (Table 2 and Table 3). Individual discussion of these mutations is presented in the following sections.

*C2689275T and G2689278A in 16srRNA Copy E* 

Two of the 5 mutations were found in the 16s ribosomal RNA (rRNA) copy E. Interestingly, both mutations were found adjacent to each other located in the 3′ major domain [47,48]. Both of the mutations are transition mutations and, therefore, less disruptive than transversion mutations. However, they still can induce functional changes since the 3′ major domain of rRNA together with the associated ribosomal proteins builds the head of the small ribosomal subunit, which is the most susceptible region for disruption by nucleotide modifications [48]. On a structural level, the mutation G2689278A, which emerged in ALE_2, might have a stronger effect on the rRNA structure due to the exchange of the strong GC pair than the mutation C2689275T found in ALE_1. The strain *C. glutamicum* gXPΔ*iolR* harbors a rearranged sequence upstream of the *xylA* open reading frame, which also altered the predicted ribosome binding site [20]. It is possible that the mutations in the major 3′ domain of the 16S rRNA copy E described in this study represent an adaptive response to the altered region upstream of the *xylA* start codon, e.g., by improving structural compatibility.

*G1992621A in cg2103 (DtxR^R103H^)* 

Interestingly, the mutation DtxR^R103H^ found in the *dtxR* gene of ALE_2, encoding the central regulator of the iron metabolism DtxR [49], was identical to the mutation found in a continuous ALE experiment to enhance growth rate in PCA-free medium supplemented with glucose [35]. The fact that the same mutation was found in two different continuous PCA-free ALE approaches supports the previous hypothesis that the cells might require enhanced iron uptake to support increased growth [35,50]. The absence of the iron chelator PCA, which typically enhances the solubility and bioavailability of ferric iron (Fe^3+^) [51], likely induced iron limitation conditions. DtxR serves as the master regulator of iron-dependent gene expression in *C. glutamicum* [49,51,52], and therefore, the emergence of the DtxR^R103H^ mutation represents an adaptive response to compensate for iron availability constraints imposed by PCA absence. Another study also published the same mutation in DtxR, which was identified in *C. glutamicum* after ALE to increase indole tolerance. The authors suggest that this specific DtxR^R103H^ mutation affects the repressor function of DtxR [53].

*C2769644T in cg2910 (IpsA^D141N^)* 

Another mutation was found in the gene *ipsA* of ALE_2 encoding IpsA, a LacI-type transcriptional regulator [54], which acts as an inositol-dependent transcriptional activator of the *myo*-inositol phosphate synthase gene *ino1*. It is known to be involved in triggering inositol-derived cell wall biogenesis during growth in glucose [54]. Recently, it was also shown that IpsA plays a role in the regulation of xylose metabolism: xylose itself can act as an effector molecule for IpsA [14]. When xylose is bound to IpsA, the binding affinity of the latter to its cognate DNA sequences is significantly reduced. The dissociation of IpsA from its DNA targets, induced by xylose, leads to widespread effects throughout the cell’s metabolic network [14]. In the same study, among other mutations, one mutation in *ipsA* was identified during an ALE experiment. The mutated IpsA^P111S^ variant is thought to be partly responsible for the rapid xylose utilization of the emerged strain as mutated IpsA^P111S^ showed higher DNA binding affinity and greater resistance to xylose compared with the wild-type IpsA [14]. This suggests that the mutation in *ipsA* found in our study might have contributed to the accelerated xylose uptake rates of the ALE_2 population compared with the parental background *C. glutamicum* gXPΔ*iolR* and populations in earlier stages of the ALE experiment (Figure 4 and Figure 5A).

*C190823G in cg0223 (IolT1^A452P^)* 

Another mutation was a single nucleotide polymorphism in the gene *iolT1* (cg0223), which was found identically in the genome of ALE_1 and ALE_2. The mutation led to an amino acid exchange from alanine to proline in the respective product IolT1. Since IolT1 is the main uptake system for both glucose and xylose in the *ptsG*-deficient strain *C. glutamicum* gXPΔ*iolR*, the occurrence of a mutation in this gene is in accordance with the design of the study, selecting for growth advantage in medium containing these two sugars. The mutation emerged early in the continuous ALE design and was verified in ALE_1 (Table 2) after 238 h of continuous cultivation. It is likely that this mutation was responsible for the sudden drop in extracellular glucose and xylose after around 150 h of continuous ALE (Figure 3).

AlphaFold analysis of IolT1 (UniProt ID: Q8NTX0) provided crucial insights into the structural basis of the observed phenotypic effect. The protein structure prediction revealed that the mutation is located in the last of 12 transmembrane alpha helices of the protein. Inducing the structurally rigid amino acid proline that lacks the NH group needed for maintaining the alpha helix backbone might have caused helix breaking [55]. The introduction of this structural disruption likely alters the protein’s conformational dynamics, thereby affecting transport kinetics. Supporting the structural significance of this position, BLAST (version 2.15.0) analysis followed by alignment of the first 25 results of the BLAST analysis in UniProt showed that the alanine residue at the respective position is highly conserved across diverse species. This underscores the potential functional and structural significance of the amino acid alanine in this position. Given that proline is known to disrupt alpha helical structures [55] and considering the central role of IolT1 in sugar uptake, this structural insight provided a compelling rationale for reverse-engineering the IolT1^A452P^ mutation into the parental background to validate its functional impact.

The resulting strain *C. glutamicum* gXPΔ*iolR* mut*iolT1* was characterized. Shake flask experiments were performed to compare the parental background strain *C. glutamicum* gXPΔ*iolR* with the evolved ALE_1 strain and the reverse-engineered strain *C. glutamicum* gXPΔ*iolR* mut*iolT1*. The latter exhibited comparable growth kinetics and sugar uptake kinetics to the population ALE_1 (Figure 6 and Table 3). The growth rate and both the xylose and glucose uptake rates of the two strains were significantly higher than in the parental background *C. glutamicum* gXPΔ*iolR*. The final biomass concentrations of both *C. glutamicum* gXPΔ*iolR* mut*iolT1* and ALE_1 were lower than that of the parental strain, indicating imbalanced metabolic flux and by-product formation. Indeed, HPLC analysis showed production of the organic acids succinate, acetate, and lactate in ALE_1 and *C. glutamicum* gXPΔ*iolR* mut*iolT1*. Since only the step of uptake of the sugars is enhanced, further engineering is required to balance the carbon flux inside of the cells and prevent by-product formation. Different studies were published to enhance substrate uptake via IolT1 (and IolT2). In contrast with this work, the binding site of *iolR* in the promotor regions of *iolT* was mutated [5,15,24] instead of deleting *iolR*. To our knowledge, this is the first study publishing a single mutation in *iolT1* that significantly enhances PTS-independent substrate uptake and growth rate in *C. glutamicum* with deletions in *ptsG* and *iolR*.

## 5. Conclusions

Taken together, by combining rational strain engineering with continuous ALE, a *ptsG*-independent *C. glutamicum* strain was constructed that efficiently co-utilizes glucose and xylose. Furthermore, a single mutation in the gene *iolT1* was found that is responsible for boosting the growth and uptake rates of glucose and xylose in *C. glutamicum*. These results represent promising starting points for the construction of production strains that require PEP as a precursor using mixed carbon source media, e.g., lignocellulosic hydrolysates as a carbon source.

## Figures and Tables

**Table 1:** Bacterial strains used within this work.

Strain	Relevant Characteristics	References
*E. coli* DH5α	F-Φ80*lacZ*ΔM15 Δ(*lacZYA-argF*) U169 *endA1 recA1 hsdR17* (rK^−^, mK^+^) *supE44 thi-1 gyrA96 relA1 phoA*	[26]
*E. coli* S17-1	*recA pro hsdR* RP4-2-Tc::Mu-Km::Tn7	[27]
*C. glutamicum* WT	*C. glutamicum* wild type (WT), ATCC13032	[28,29]
*C. glutamicum* gX	*C. glutamicum* Δ*actA*::*xylAB* (*C. glutamicum* WT derivate with the synthetic operon, consisting of *xylA* of *Xanthomonas campestris* and *xylB* of *C. glutamicum* WT, integrated into the gene locus *actA* (cg2840) with evolved sequence upstream of *xylA*)	[20]
*C. glutamicum* gXP	*C. glutamicum* gX with the deletion of gene *ptsG* encoding PTS system glucose-specific EIICBA component	This study
*C. glutamicum* gXPΔ*iolR*	*C. glutamicum* gXP with the deletion of gene *iolR* encoding the transcriptional regulator IolR	This study
*C. glutamicum* ALE_1	Population of evolved *C. glutamicum* gXPΔ*iolR* after stage 1 (300 h of continuous adaptation)	This study
*C. glutamicum* ALE_2	Population of evolved *C. glutamicum* gXPΔ*iolR* after stage 2 (600 h of continuous adaptation)	This study
*C. glutamicum* gXPΔ*iolR*Δ*iolT1*	*C. glutamicum* gXPΔ*iolR* with the deletion of gene *iolT1*, encoding *myo*-inositol/proton symporter IolT1	This study
*C. glutamicum* gXPΔ*iolR*Δ*iolT1*::mut *iolT1*	*C. glutamicum* gXPΔ*iolR*Δ*iolT1* with integrated mut*iolT1* (*iolT1*^A452P^), encoding *myo*-inositol/proton symporter IolT1	This study

**Table 2:** Mutations found in the population ALE_1 after 238 h of continuous ALE. Nucleotide substitutions are indicated with genomic position, along with the resulting amino acid changes at the corresponding protein positions.

Gene	Mutation	Frequency [%]
*iolT1* (cg0223)	C190823G → A452P	99.86
16s rRNA copy E	C2689275T	100.00

**Table 3:** Mutations found in the population ALE_2 after 600 h of continuous ALE. Nucleotide substitutions are indicated with genomic position, along with the resulting amino acid changes at the corresponding protein positions.

Gene	Mutation	Frequency [%]
*iolT1* (cg0223)	C190823G → A452P	99.86
16s rRNA copy E	C2689275T	90.91
16s rRNA copy E	G2689278A	90.91
*dtxR* (cg2103)	G1992621A → R103H	97.66
*ipsA* (cg2910)	C2769644T → D141N	97.46

**Table 4:** Comparison of maximal growth rate (µ_max_), biomass specific glucose (q_glc_), and xylose (q_xyl_) uptake rate of parental *C. glutamicum* gXPΔ*iolR*, evolved *C. glutamicum* gXPΔ*iolR* (ALE_1), and reverse-engineered *C. glutamicum* gXPΔ*iolR* (mut*iolT1*) strain. Data represent means of 3 replicates with standard deviations. Significance was determined based on a two-sided unpaired Student’s *t*-test (*** *p* ≤ 0.001, * *p* ≤ 0.05).

*C. glutamicum* gXPΔ*iolR* Strain	µ_max_ [h^−1^]	q_glc_ [g g^−1^ h^−1^]	q_xyl_ [g g^−1^ h^−1^]
parental	0.20 ± 0.00 ***	0.36 ± 0.02 ***	0.15 ± 0.01 *
ALE_1	0.27 ± 0.01	0.68 ± 0.03	0.18 ± 0.01
mut*iolT1*	0.27 ± 0.01	0.66 ± 0.04	0.18 ± 0.01

## Data Availability

The raw data supporting the conclusions of this article are available in a publicly accessible repository at https://doi.org/10.18419/DARUS-5107.

## Supplementary Materials

The following supporting information can be downloaded at https://www.mdpi.com/article/10.3390/microorganisms13071606/s1: Table S1: Plasmids used in this study; Table S2: Oligonucleotides used in this study; Table S3: Equations used for the calculation of kinetic parameters; Figure S1: Schematic overview of the ALE process.

## Abbreviations

The following abbreviations are used in this manuscript:

ALE	adaptive laboratory evolution
*C. glutamicum*	*Corynebacterium glutamicum*
DO	dissolved oxygen
dYT	double yeast tryptone
*E. coli*	*Escherichia coli*
glc	glucose
GRAS	generally recognized as safe
LB	lysogeny broth
MOPS	3-(N-morpholino)propanesulfonic acid
OD	optical density
PCA	protocatechuic acid
PEP	phosphoenolpyruvate
PTS	phosphotransferase system
WGS	whole-genome sequencing
XI	xylose isomerase
xyl	xylose
