# Supplementary material for: Single Mutation in iolT1 in ptsG-Deficient Corynebacterium glutamicum Enables Growth Boost in Xylose-Containing Media
Source: Microorganisms. 2025 Jul 8;13(7):1606. doi: 10.3390/microorganisms13071606 (PMC12298647; doi:10.3390/microorganisms13071606)
Supplement: Supplementary file 1 [file microorganisms-13-01606-s001.zip › microorganisms-3714467-supplementary.pdf]

Supplementary Table S1: Plasmids used in this study.

Plasmids		
pK19*mobsacB*	Km[r]; Mobilizable cloning vector to construct insertion or deletion mutants of *C. glutamicum* (pK18 *ori*$V_{E.c.}$ *sacB*, *lacZα*)	[33]
pK19*mobsacB*-Del-*iolT*1	pK19*mobsacB* with *iolT*1 deletion construct	study
pK19*mobsacB*-inte-mut*iolT*1	pK19*mobsacB* with mut*iolT*1 integration construct	study
pK19*mobsacB*Δ*iolR*	pK19*mobsacB* with *iolR* deletion construct	study
pK19*mobsacB*Δ*ptsG*	pK19*mobsacB* with *ptsG* deletion construct	study

Supplementary Table S2: Oligonucleotides used in this study.

Primer	Sequence (5' → 3')	Purpose
ptsG-UF-fw	CATGCCTGCAGGTCGACTCTAGAGATGGCGTCCAAACTGACGACG	Amplification of *ptsG* upstream fragment for Gibson
ptsG-UF-rv	CGTTGTAAAACGACGGCCAGTGTTACTCGTTCTTGCCGTTGACCTTGATC	Amplification of *ptsG* upstream fragment for Gibson
ptsG-DF-fw	TACCTCCTTTATTCTTTCCGAACCATTATAACACAGATTCAAATTAATGTCAATTATGGCAGGAAGTAGAAGACCGAGC	Amplification of *ptsG* downstream fragment for Gibson
ptsG-DF-rv	GCGAAGGCATATTACGGGCAGTAAACCAGGCATTGCAATCCAACC	Amplification of *ptsG* downstream fragment for Gibson
ptsG-UF-g	GTTGTGAGGCTTGGTTTTCGTACGG	Verification of *ptsG* deletion
ptsG-DF-g	GGCACTTGAGAAGCGATTCGACG	Verification of *ptsG* deletion

iolR-UF-fw	CGCCAAGCTTGCATGCCTGCAG GTCGACTCTAGAGGAGGTACTT GCCGAAAGATTG	Amplification of *iolR* upstream fragment for Gibson
iolR-UF-rv	CAACTCGATTACTTGGCCGGAG GCTACTTGGAAGTAGAGGGG	Amplification of *iolR* upstream fragment for Gibson
iolR-DF-fw	CCCCTCTACTTCCAAGTAGCCT CCGGCCAAGTAATCGAGTTG	Amplification of *iolR* downstream fragment for Gibson
iolR-DF-rv	CGACGGCCAGTGAATTCGAGCT CGGTACCCGGGGCCATTCCAG TAGGCGCTGAGG	Amplification of *iolR* downstream fragment for Gibson
iolR-UF-g	GATCGGTGGCAACGTACTGG	Verification of *iolR* deletion
iolR-DF-g	CGTTGTCATGTACGGTCTGGC	Verification of *iolR* deletion
iolT1-UF-fw	ACCATTCCCGATGTCCGCTC	Amplification of *iolt1* upstream fragment for Gibson
iolT1-UF-rv	*TTAGTGCACCTTTCCGAAGGTA CTAGCCAT*CTTGT	Amplification of *iolt1* upstream fragment for Gibson
iolT1-DF-fw	*ATGGCTAGTACCTTCGGAAAGG TGCACTAA*AAACC	Amplification of *iolt1* downstream fragment for Gibson
iolT1-DF-rv	CCACTTCTATTCGCAAGAG	Amplification of *iolt1* downstream fragment for Gibson
iolT1-UF-g	ACTGCAGCGGATGAGTTTG	Verification of *iolT1* deletion or mut*iolT1* integration
iolT1-DF-g	TCACATTCGCCCCACCAGT	Verification of *iolT1* deletion or mut*iolT1* integration

Supplementary Table S3: Equations used for calculation of kinetic parameters.

Growth rate μ in h^{-1} during batch phase	$\mu = \ln\left(\frac{c_{x,2}}{c_{x,1}}\right)\frac{1}{t_2 - t_1}$
Growth rate μ in h^{-1} during continuous phase	$\mu = D$

Biomass-substrate yield Y_{XS} in gg^{-1} (steady state concentrations used during continuous phase)	$$Y_{XS} = \frac{c_{x,2} - c_{x,1}}{c_{S,1} - c_{S,2}}$$
Biomass specific uptake rates q_S in $gg^{-1}h^{-1}$ (steady state concentrations used during continuous phase)	$$q_S = \mu \frac{c_{S,1} - c_{S,2}}{c_{x,2} - c_{x,1}}$$

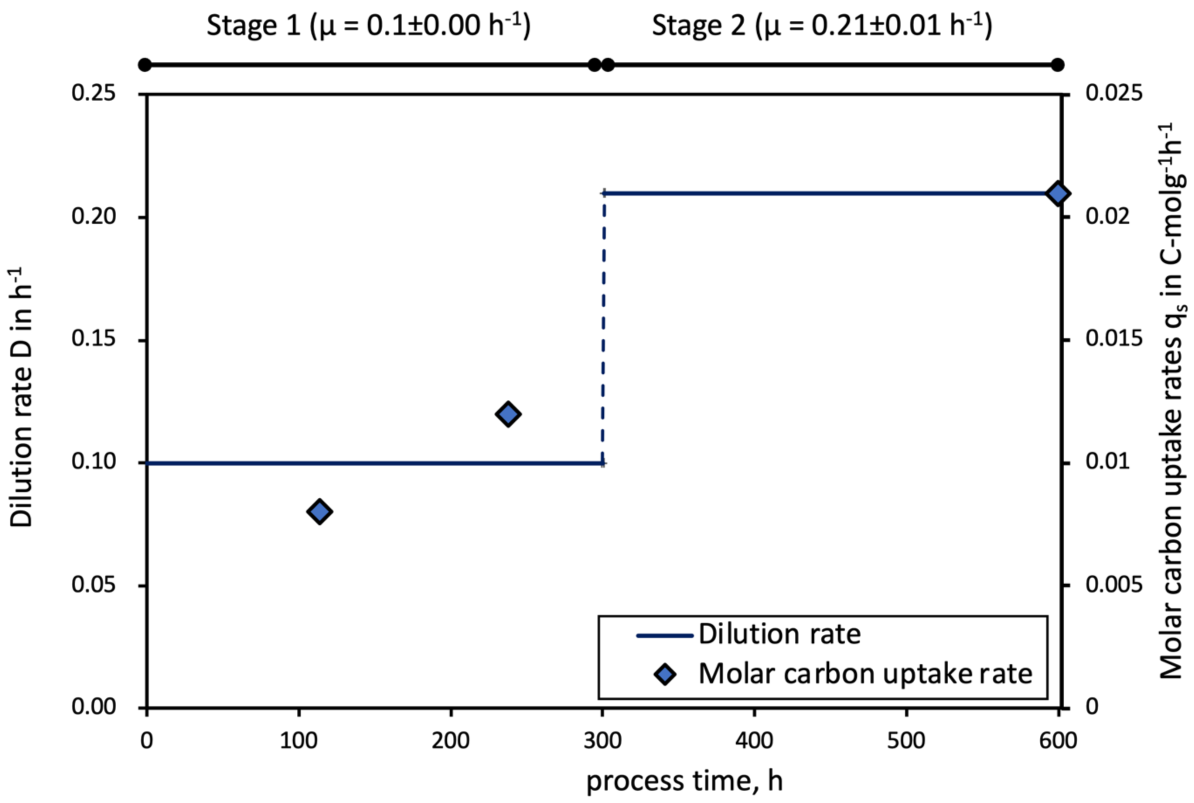

Supplementary Figure S1: Schematic overview of the ALE process. Two consecutive continuous cultivations, each lasting 300 h, were conducted at different dilution rates using medium containing the two carbon sources glucose and xylose. The first stage was conducted with D set to 0.1±0.00 h^{-1} and the second stage with D set to 0.21±0.01 h^{-1}. The population response was an increase in sugar uptake rates for both glucose and xylose. Biomass specific molar carbon uptake rates are shown as representative examples.
